# Effect of pluronic block polymers and N-acetylcysteine culture media additives on growth rate and fatty acid composition of six marine microalgae species

**DOI:** 10.1007/s00253-021-11147-8

**Published:** 2021-02-12

**Authors:** Justine Sauvage, Gary H. Wikfors, Xiaoxu Li, Mark Gluis, Nancy Nevejan, Koen Sabbe, Alyssa Joyce

**Affiliations:** 1 Department of Marine Sciences, University of Gothenburg, Gothenburg, Sweden; 2 NOAA Fisheries Service (NMFS) Northeast Fisheries Science Center, Milford, CT 06460 USA; 3 South Australian Research and Development Institute, Primary Industries and Regions (PIRSA) Aquatic Sciences Centre, West Beach, SA Australia; 4 Laboratory of Aquaculture & Artemia Reference Center, Faculty of Bioscience Engineering, Ghent University, Ghent, Belgium; 5 Laboratory of Protistology & Aquatic Ecology, Faculty of Sciences, Ghent University, Ghent, Belgium

**Keywords:** Marine microalgae, Growth medium formulation, Biochemical composition, Live (aquaculture) feed, Pluronic block polymers, N-acetylcysteine

## Abstract

**Abstract:**

The efficiency of microalgal biomass production is a determining factor for the economic competitiveness of microalgae-based industries. N-acetylcysteine (NAC) and pluronic block polymers are two compounds of interest as novel culture media constituents because of their respective protective properties against oxidative stress and shear-stress-induced cell damage. Here we quantify the effect of NAC and two pluronic (F127 and F68) culture media additives upon the culture productivity of six marine microalgal species of relevance to the aquaculture industry (four diatoms-*Chaetoceros calcitrans*, *Chaetoceros muelleri*, *Skeletonema costatum*, and *Thalassiosira pseudonana*; two haptophytes-*Tisochrysis lutea* and *Pavlova salina*). Algal culture performance in response to the addition of NAC and pluronic, singly or combined, is dosage- and species-dependent. Combined NAC and pluronic F127 algal culture media additives resulted in specific growth rate increases of 38%, 16%, and 24% for *C. calcitrans*, *C. muelleri*, and *P. salina*, respectively. Enhanced culture productivity for strains belonging to the genus *Chaetoceros* was paired with an ~27% increase in stationary-phase cell density. For some of the species examined, culture media enrichments with NAC and pluronic resulted in increased omega-3-fatty acid content of the algal biomass. Larval development (i.e., growth and survival) of the Pacific oyster (*Crassostrea gigas*) was not changed when fed a mixture of microalgae grown in NAC- and F127-supplemented culture medium. Based upon these results, we propose that culture media enrichment with NAC and pluronic F127 is an effective and easily adopted approach to increase algal productivity and enhance the nutritional quality of marine microalgal strains commonly cultured for live-feed applications in aquaculture.

**Key points:**

*• Single and combined NAC and pluronic F127 culture media supplementation significantly enhanced the productivity of Chaetoceros calcitrans and Chaetoceros muelleri cultures.*

*• Culture media enrichments with NAC and F127 can increase omega-3-fatty acid content of algal biomass.*

*• Microalgae grown in NAC- and pluronic F127-supplemented culture media are suitable for live-feed applications.*

**Supplementary Information:**

The online version contains supplementary material available at 10.1007/s00253-021-11147-8.

## Introduction

Microalgae are commercially relevant for a wide variety of biotechnology applications because of rapid growth rates and synthesis of large amounts of macromolecules (e.g., carbohydrates, lipids, and proteins) and other chemicals [e.g., polyunsaturated fatty acids, pigments, vitamins, polysaccharides, antioxidants, and other bioactive metabolites] (Bleakley and Hayes 2017; Borowitzka 1988; Brennan and Owende 2010; Chew et al. 2017; Liu et al. 2016; Nicoletti 2016; Wijffels et al. 2010). Cultured microalgae are essential as live feeds for the commercial rearing of many aquatic animals, especially larval bivalve molluscs, crustaceans, and zooplankton (Guedes and Malcata 2012; Wikfors et al. 1992). Mass-cultured microalgae are regarded as a promising resource for the development of novel aquafeeds given nutritional profiles rich in high-quality proteins, vitamins, micronutrients, and carotenoids (Muller-Feuga 2000; Rahman 2020; Shah et al. 2018; Yaakob et al. 2014).

Most aquaculture hatcheries have microalgae production facilities in-house for the production of live feed (Guedes and Malcata 2012). Efficiency of hatchery microalgal production is dependent upon the ability to achieve high biomass densities in strains with high nutritional value and rapid growth rates. High production costs and challenges with scalability of current mass-culturing and harvesting technologies represent major constraints to the expansion of the aquaculture industry (Guedes and Malcata 2012; Khan et al. 2018). For example, algae production remains a major component of the operating cost of shellfish hatcheries (i.e., 30–40% of total operating cost, attributable to high labor, water and energy requirements) and a limiting factor for overall hatchery production output (Kaspar et al. 2014; Persoone and Claus 1980). Solutions to consistently and reliably boost algal growth, in a cost-effective and easily implementable manner, as well as technological innovations to increase the scale of production, are necessary for expansion of the aquaculture and aquafeed industries (Oostlander et al. 2020).

Environmental culture conditions (i.e., light, temperature, CO_2_ concentration, nutrients, salinity, pH, and mixing conditions) and the choice of cultivation system (open *versus* closed systems, photobioreactors) affect the efficiency and biomass composition of microalgae (Singh and Dhar 2011). Current culture media formulations for high-density cultivation of marine microalgae consist of seawater enrichments with macronutrients (N,P), chelated trace metals, and in some cases vitamins, at levels far exceeding those found in natural aquatic environments (Guillard and Ryther 1962). In addition to the comprehensive suite of macro-and-micro-nutrient inputs, supplementation of the culture media with other chemical and/or biological agents that modify culture conditions, or alter algal physiology, represents an opportunity to achieve optimal growth rates and culture performance. Similar approaches have been implemented successfully in soil-based agriculture, wherein in addition to fertilizers, the application of exogenous chemical compounds (e.g., biostimulants) or use of plant growth-promoting microorganisms is routinely introduced to stimulate productivity and enhance crop resistance to biotic and abiotic stressors (Bartelme et al. 2018; Mueller and Sachs 2015; Rouphael and Colla 2018; Yakhin et al. 2017). In the present study, we propose two novel culture media additives with the capacity to alleviate cellular oxidative and shear-stress-induced cell damage, two key processes known to impede overall culture performance in microalgal cultivation systems (Rijstenbil 2003; Wang and Lan 2018; Xie et al. 2019).

In aerobic environments, reactive oxygen species (ROS) and free radicals are generated continuously as a result of normal, intracellular metabolism (photosynthesis) as well as external factors (e.g., light, nutrient limitation). Cellular ROS accumulation causes oxidative stress, a state of imbalance between the production of ROS and the neutralization of free radicals by antioxidants, resulting in a disruption of redox signaling and progressive breakdown of biomolecules (Cirulis et al. 2013; Xie et al. 2019). N-acetylcysteine (NAC) is a thiol-containing compound utilized both experimentally and clinically to reduce glutathione (GSH). The redox-active thiol group enables NAC and GSH to scavenge oxidants (e.g., ROS and free radicals), acting as a redox buffer, thereby contributing to the maintenance of cellular homeostasis under normal and stress conditions (Cotgreave 1996; Dröge et al. 1992; Roederer et al. 1992; Schafer and Buettner 2001; Sen 2001). Further, GSH is associated with numerous physiological functions in plants including carbon, nitrogen, and sulfur metabolism, regulation of growth and development, cell defense, redox signaling, and regulation of gene expression (Aw 1999; Cai and Jones 1998; Foyer et al. 1997; Foyer and Noctor 2009; Kirlin et al. 1999; May et al. 1998). The low toxicity of NAC, solubility in water, ability to cross biological membranes, combined with its multifaceted roles observed in plant growth experiments in response to environmental and redox stressors, offers a promising approach for the enhancement of cellular antioxidant defense in microalgal production systems (Cotgreave 1996; Dröge et al. 1992; Gillissen et al. 1997; Noctor et al. 1998; Noctor and Foyer 1998; Sandstrom et al. 1994).

Few studies have evaluated the potential of NAC applied topically to plants (e.g., rice and soybean); other studies have investigated NAC effects when included in the culture medium of a microalga, *Chlorella vulgaris*, as a strategy for controlling pathogenic bacteria and minimizing oxidative damage (Foyer and Noctor 2009; Goldstein 2008; Malanga et al. 1999; Munirah et al. 2015; Muranaka et al. 2013; Nozulaidi et al. 2015). Effects of NAC supplementation on the growth performance of marine phytoplankton species, however, remain unexplored.

Similarly, management of shear stress is an important consideration for the high-output cultivation of marine microalgal strains. Culture sparging and stirring generate hydrodynamic forces that act on microalgal cell membranes, leading to cell mortality and decreased population growth rate (Rodríguez et al. 2009; Wang and Lan 2018).

Pluronic block copolymers (also known under the non-proprietary name “poloxamers”) are a group of polymer nanomaterials consisting of hydrophilic poly(ethylene oxide) and hydrophobic poly(propylene oxide) blocks routinely used in pharmaceutical research as vehicles for chaperoning therapeutic agents into cells (Batrakova and Kabanov 2002). The amphiphilic nature of these copolymers imparts surfactant properties, including the ability to interact with hydrophobic surfaces and biological membranes (Kabanov et al. 2002). The surfactant properties of pluronics can protect cells from the shear-stress-induced damage that occurs to mammalian (Michaels et al. 1991; Tharmalingam et al. 2008), insect (Wu et al. 1997), marine sponge (Camacho et al. 2005), and microalgal cell cultures [i.e., diatom *Phaeodactylum tricornutum*] (Mirón et al. 2003; Rodríguez et al. 2009). The mechanisms involved in the alleviation of shear-stress-induced cellular damage in the presence of pluronic compounds include the following: (i) decreased bubble-cell attachment, (ii) decreased surface tension, and (iii) decreased energy generated from bubble bursting (Dey et al. 1997; Jordan et al. 1994; Ma et al. 2004; Michaels et al. 1995). Furthermore, recent developments indicate that selected pluronic block polymers act as biological-response-modifying agents, having the ability to regulate a variety of cell processes such as ATP synthesis, gene expression, and apoptotic signal transduction (Kabanov 2006; Kabanov et al. 2005). As such, we infer that the cell surfactant properties-in combination with the biological response modifying capabilities of pluronic block polymers - are relevant to optimize microalgae cultivation practices in large-scale photobioreactors.

In the present study, we evaluated if supplementation of N-acetylcysteine and pluronic block polymers (Pluronic ® F127 and F68) to the culture media have beneficial effects upon the culture performance of marine microalgal strains produced as live feeds for the aquaculture industry (Guedes and Malcata 2012). Culture media enrichment effects upon algal biomass fatty acid composition and algal release of transparent exopolymer particles (TEP), two key indicators for the production of high-quality live feeds, were also examined.

Our results show that NAC and pluronic medium additives can have significant effects upon algal yield and algal biomass unsaturated fatty acid composition, but these effects differ significantly between species. Effects observed are interpreted in the light of known mechanisms underlying the actions of NAC and pluronic in illuminated and sparged bioreactors. Finally, by preforming a trial in an oyster hatchery, we evaluated the applicability of these novel algal media constituents for use in live feed production by performing a feeding trial with *Crassostrea gigas*, a commercially important bivalve species contributing ~10% of worldwide mollusc production (FAO 2015)

## Materials and methods

### Microalgae and growth medium

Bacteria-free *Chaetoceros calcitrans* (Chaet cal.) and *Tisochrysis lutea* (T-Iso) stock cultures were obtained from the NOAA Milford Laboratory Microalgal Culture Collection, Northeast Fisheries Science Center, Milford, USA. Axenic *Chaetoceros calcitrans* (CS-178), *Chaetoceros muelleri* (CS-176), *Thalassiosira pseudonana* (CS-173), *Skeletonema costatum* (CS-252), *Tisochrysis lutea* (CS-177), and *Pavlova salina* (CS-49) stock cultures were sourced from the Australian National Algae Culture Collection, CSIRO, Hobart, Australia. The selected microalgal species are cultivated commonly in aquaculture hatcheries as live feeds. Stock cultures were transferred into 250-mL Erlenmeyer flasks filled with 100-mL 1-μm filtered, autoclave sterilized, F nutrient-enriched seawater (Guillard and Ryther 1962). Starter cultures were kept at 20°C under continuous illumination, gently swirled daily, and maintained by performing re-inoculations in fresh medium every 14 days. Prior the start of the experiment, starter cultures used as inocula were confirmed to be bacteria-free through bacterial-DNA-specific staining of a culture subsample using the SYTO 9 nucleic acid stain (*ThermoFisher*). Stained bacterial cells were enumerated by flow cytometry (*BD Accuri C6*). Algal cultures were considered bacteria-free and adequate for experimental work if event counts were below 100 flow-cytometer events within the bacterial gate (considered to be electronic noise events) per milliliter of sample analyzed.

### Treatments and reagents

A stock solution (300 mM) of N-acetyl-L cysteine (NAC, >98% purity, A15409, *Alfa Aesar*) was prepared in autoclave-sterilized, Milli-Q grade water. NAC culture media enrichments were designed to achieve a final concentration of 0.1, 1, and 5 mM in the experimental flasks. The experimental NAC concentration range was selected based upon prior experimental work from Malanga et al. (1999), wherein *Chlorella vulgaris* cultures were supplemented with 1–5 mM NAC as protection against UV-B-related damage.

Two pluronic block polymers (F127 and F68) with different physiochemical properties were examined as culture medium additives. The F127 pluronic block polymer, also referred to under the trademark “Pluronic® F127,” was purchased from *Sigma* (P2443). This compound has a molecular weight of 12,600 g mol^−1^, is water soluble with a 0.7% (w/v) critical micellar concentration at 25°C, and is composed of 65 PPO and 200 PEO units (hydrophilic-lipophilic balance, HLB, 18-23). The relatively less-hydrophobic Pluronic® F68 was obtained from *ThermoFisher* [24040032]. Pluronic F68 has a molecular weight of 8400 g mol^−1^, is equally soluble in water with 2.6% (w/v) critical micellar concentration at 25°C, and is composed of 43 PPO and 38 PEO units [HLB > 24] (Alexandridis and Hatton 1995; Bodratti and Alexandridis 2018). To avoid excessive foaming and micelle formation in the algal cultures resulting from pluronic culture media enrichments, F68 and F127 experimental dosages were kept below critical micellar concentrations, which ranged between 0.1–0.001% and 0.01–0.005% (w/v), respectively. Sterilized milli-Q grade water was used for the preparation of all solutions. To suppress foaming in the algal cultures following pluronic block polymer enrichment, especially in the case of bubbled flasks, an antifoam (Antifoam A – *Sigma*) additive was included in a selection of treatments. Preliminary experiments determined that 10^−4^% (w/v) antifoam concentration in the culture medium effectively suppressed foaming while not inhibiting algal growth for the six microalgal strains targeted in this study.

### Experimental design and culture conditions

A series of NAC, pluronic block polymer, and antifoam treatments (i.e., culture media additives), singly or combined, was formulated and tested for the cultivation of marine microalgal species in static (i.e., non-sparged) and bubbled experimental flasks. In an initial screening, the effect of culture media supplementation with NAC or pluronic (F127 and F68) on culture performance (i.e., specific growth rate and final cell density) of the diatom *Chaetoceros calcitrans* and the flagellate *Tisochrysis lutea* was assessed in static, batch-culture experiments. Subsequently, a second microalgal cultivation experiment was performed with six marine microalgal strains grown in laboratory-scale, bubbled photobioreactors. Microalgal culture conditions commonly applied for the production of live feed in aquaculture hatcheries (i.e., nutrient replete conditions, constant illumination, air-mixing, and CO_2_ supplemented air inflow) were adopted in this experiment (Helm 2004). Culture media enrichment effects upon specific growth rate, final cell density, algal TEP release, and algal biomass fatty acid composition — key variables for the production of high-quality live feeds-were assessed for each treatment and algal strain. A total of 25 culture media enrichment experimental treatments were formulated (electronic supplemental material-Table S1) to (i) assess the effect of NAC, pluronic block polymers [F127 and F68], singly and combined, upon algal culture performance; (ii) evaluate if the addition of antifoam is effective at minimizing pluronic-induced foaming with no detrimental effect upon algal growth; and (iii) constrain optimal dosage range for each additive. An overview of culture media enrichment, including single and combined NAC and pluronic additives at varying dosages (total of 25 treatments) performed in this study, is given in the electronic supplemental material-Table S1. Treatment effects were assessed relative to the culture performance and algal biomass fatty-acid composition of microalgae grown in standard, F-enriched seawater medium [hereafter referred to as *control* treatment] (Guillard and Ryther 1962).

Parallel microalgal batch culture experiments were performed at the NOAA Milford Laboratory and the South Australian Research and Development Institute (SARDI-PIRSA) under static and bubbled culture conditions, respectively. NAC and pluronic culture medium additives were added aseptically as a single dose prior to inoculation of the culture flasks.

Static, batch-culture experiments were performed in 2.8-L Fernbach flasks, filled with 650-mL F-enriched seawater medium and inoculated with 100-mL microalgal starter culture. Milford harbor water (26-28 ppt salinity, pH 7.9) was used for culture media preparation following sequential filtering through wound cotton cartridge filters (down to 1-μm mesh size) and autoclave sterilization. Cultures were grown at 18.5°C, under constant illumination. The artificial light source consisted of 1.2-m, T8 35-W, 4100 K, *GE Ecolux* with Starcoat F32T8 SPP4 bulbs located 30 cm from the culture flaks illuminating one side of the cultures at a PAR light intensity of 140 μmol photons m^−2^ s^−1^, measured at the surface of the flasks with a *Licor* Inc., Quantum/Radiometer/Photometer (Model LI-185B). Experimental flasks were swirled daily to remobilize cells and promote gas exchange.

Bubbled photobioreactors consisted of 250-mL Erlenmeyer flasks, each fitted with a rubber stopper with two holes serving as the gas inlet and outlet. Air at a flow rate of 0.4 L min^−1^ with 0.5% CO_2_/air mixture was sparged from the bottom of the flask by a glass distribution tube that was inserted into the photobioreactor through one of the holes in the cap. LED lamps were used to illuminate the photobioreactors from one side of the flasks with a continuous light intensity of 140 μmol photons m^−2^ s^−1^ measured at the surface of the flasks. The total culture volume in the reactors was 150 mL, consisting of autoclave-sterilized, F-enriched seawater medium (30 ppt, pH 8), and algal inoculum (10–20 mL). The temperature of the reactors was maintained at 20.5°C. The CO_2_/air mixture ratio was increased gradually during algal culture development to maintain the desired culture pH of 8.2.

Initial cell counts ranged between 10^4^ and 10^5^ cells mL^−1^ for static and bubbled experiments, depending upon algal species. Microalgal culture development was monitored daily throughout the course of the experiment by aseptically extracting a 200-μL culture subsample followed by the enumeration of microalgal cells by flow cytometry (*BD Accuri C6*). Experiments were terminated after confirmation that the cultures had reached stationary phase for two consecutive sampling points (i.e., ~ 8 to 14 days for bubbled and static experiments, respectively). Prior to termination of the experiments, the absence of bacteria was confirmed through bacterial DNA specific staining (SYTO 9) of a culture aliquot followed by flow cytometric enumeration of stained particles. Key algal culture variables (i.e., temperature, light, CO_2_ flow, and pH) were monitored periodically throughout the course of the experiment for both the static and bubbled culture experiments.

### Analyses

Microalgae culture subsamples (5 mL) from each treatment and replicate were harvested immediately following inoculation and every 24 or 48 h for bubbled or static cultures, respectively. Culture samples were collected aseptically in a transfer hood and placed in a sterile falcon tube for measurement of pH and cell density. A 10-μL aliquot was removed for a 10-fold dilution in filter-sterilized (0.22-μm mesh size) seawater for algal cell count determination by flow cytometry. The relative standard error for 10 replicate particle counts was < 8%. After reaching stationary phase, a 100-mL algal culture aliquot was centrifuged, and the resulting algal biomass pellet was used for the characterization of polyunsaturated fatty acid composition. Samples were freeze-dried and stored at −80°C for later analysis. Lastly, prior to termination of the algal culture experiments, a 2-mL culture aliquot was extracted to quantify microalgal culture EPS abundance at stationary phase.

### Fatty acid profiles

Fatty acid methyl esters (FAMEs) were extracted using a modification of the Lepage et al. (1984) method (Lepage and Roy 1984). A methanol/toluene (3:2 v/v-5 mL) mixture, acetylchloride/methanol (1:20 v/v-5 mL) mixture, and internal standard solution [containing 4.78 mg ml^−1^ 20:2 (*n*-6) fatty acid dissolved in iso-octane] was added to an ~100-mg freeze dried and homogenized microalgal powder in a glass tube with Teflon cap. Tubes were placed in a hot-water bath (100°C) for 1 h and shaken regularly. Thereafter, cool, distilled water (5 mL) and hexane (5 mL) were added for phase separation and extraction. Tubes were centrifuged for 5 min (1300×*g*), after which the upper (organic) phase was collected in a separate glass container. The lower, remaining aqueous phase was subsequently reextracted two additional times using only hexane for phase separation. Combined organic phases were dried by vacuum filtering through a P3 filter partly filled with anhydrous sodium sulfate powder followed by solvent evaporation on a rotary evaporator at 35°C. Extracted lipids (FAMEs) were dissolved in 0.5-mL iso-octane and stored until analysis. FAME distributions and concentrations were measured through gas chromatography using an *Agilent 7890B gas chromatograph* equipped with a polar capillary column, BPX70 (*forte series*, *SGE Australia*, 50 m, 0.32-mm diameter, 0.25-μm film thickness). Per analysis, a 20-μL subsample of lipid extract diluted in iso-octane was injected into the instrument. Hydrogen was used as the carrier gas at a constant flow of 3.1 mL min^−1^. Samples were injected at an oven temperature of 85°C, which was increased to 150°C at a rate of 30°C min^−1^. The temperature conditions were further adjusted past 150°C: increase at 0.1 °C min^−1^ from 150 to 152°C, from 152 to 172°C at 0.5°C min^−1^, and from 172 to 200°C at 50°C min^−1^ and held for 7 min. Standard reference FAME mixtures (GLC-68 series, *Nu-Chek-Prep*, Inc., USA) were used for FAME peak identification on the chromatograms. Individual FAME concentrations were quantified relative to the concentration of the internal standard.

### Transparent exopolymer particle concentration

Transparent exopolymeric particle (TEP) concentrations were determined according to the dye-binding assay technique developed by Passow and Alldredge (1995). Culture samples were filtered (< 130 mbar) onto 0.4-μm pore-size polycarbonate filters (nucleopore track-etched membranes, *Whatman*). The volume of algal culture sample filtered ranged from 0.5 to 2 mL, which was adjusted for each sample to avoid filter clogging. For each sample, filters were prepared in triplicate. Particles on the filter were stained for approximately 4 s with 1 ml of a 0.02% aqueous solution of alcian blue (8GX; *Sigma-Aldrich*) dissolved in 0.06% acetic acid (0.2 μm filtered, pH = 2.5). Stained filters were rinsed gently with 2-mL ammonium formate solution (0.47 M) to remove excess dye and salt, transferred into centrifuge tubes, and stored at −20°C for subsequent colorimetric determination. An ammonium formate rinse solution was chosen to avoid cell damage or cell bursting from osmotic imbalance during the excess stain removal step. The alcian blue stain was extracted from the filters in 80% sulfuric acid (3-h incubation period) and analyzed for UV absorption at 787 nm using a spectrophotometer (Cytation 3 Cell Imaging Multi-Mode Reader, *BioTek Instruments*). Given that alcian blue binds to acidic and sulfated polysaccharides, this method is semi-quantitative when the chemical composition of TEP is unknown (Passow and Alldredge 1995; Ramus 1977). Methods described in Bittar et al. (2018) were followed for the calibration of TEP measurements using a commercially available polysaccharide xanthan gum powder as reference material (Bittar et al. 2018). The concentration of TEP was determined in units of mass Gum Xanthan (X) equivalents per volume sampled, or μg X eq. L^−1^. Microalgal culture TEP abundances were quantified at stationary phase conditions for all treatments and replicates (*n*=3).

### Larval feeding trial

*Crassostrea gigas* broodstock were spawned by temperature shock (23 ± 5°C) at the South Australian Research and Development Institute (SARDI)-Primary Industries and Regions South Australia (PIRSA). Broodstock were sourced locally from two commercial farms in Coffin Bay, South Australia. Fertilized eggs were held in a static, 200-L tank for 18 h at 23°C with gentle aeration. Following this incubation period, D-stage larvae were harvested by draining the tank onto a 45-μm mesh screen, rinsed thoroughly, and transferred to 20-L conical tanks at a starting stocking density of 250 larvae mL^−1^. Larval tanks were run in a flow-through system (i.e., continuous water and food supply) according to standard hatchery practices (Supan 2014). Inflowing seawater was UV-treated, heated to 22°C, sequentially filtered, and passed through a final 0.04-μm membrane filter. The water temperature in the rearing tanks fluctuated between 23 and 25°C. Larvae were reared under these conditions and maintained for 6 days following standard FAO hatchery protocols (Helm 2004). Larval-rearing tanks were cleaned daily with chlorinated water (200 ppm), followed by freshwater and seawater rinses. During the initial 5 days of larvae development, larvae were fed an equal proportion mixture, on a per cell basis, of *Chaetoceros calcitrans*, *Pavlovo salina*, and *Tisochrysis lutea* grown in Walne’s algal medium (Walne 1970). On the sixth day of development, shellfish larvae were graded on a 100-μm sieve (average larvae shell length: 133 ± 3 μm) and transferred to six, 200-L conical tanks and reared in static conditions, gently aerated and stocked at 5 larvae mL^−1^ (i.e., 1 million larvae per tank). From this point, the shellfish diet was adjusted to a mixture of *Chaetoceros calcitrans*, *Chaetoceros muelleri*, *Pavlovo salina*, and *Tisochrysis lutea* (equal proportions based upon cell numbers) grown in F medium, representing the initiation of the feeding trial. Three replicate tanks were fed F-medium-grown algae [hereafter called *control treatment*), and three replicate tanks were fed algae grown in F medium supplemented with NAC (1 mM) and F127 pluronic block polymer (0.001%) [hereafter called *NAC + F127 treatment*]. Algal feed was grown in 16-L polyethylene carboys under identical cultivation conditions as the 250-mL photobioreactors described above. Larval-rearing tanks were supplied with algae manually twice daily to maintain algal cell densities in the rearing water at 10–30,000 cells mL^−1^. Algal consumption was monitored once a day by measuring cell densities in the rearing water prior to the next feeding using a Sedgewick-Rafter slide and microscope. The appropriate volume of algal feed for the following 24 h was adapted according to algal-feed consumption efficiency determined on the day prior. As part of routine larval grading, stocking densities were decreased through culling to maintain optimal larval density and to diminish risk of bacterial infection. For the following 10 days of larval development (from day 6 until day 16), larval densities, mortality, and size were quantified at 2–3-day intervals in triplicate. For this purpose, tanks were drained, and larvae were caught on a sieve and cleaned with a gentle seawater spray and resuspended in 500-mL seawater. Aliquots from the concentrated larval samples (1 mL) were transferred to 12-well plates for counting, and mortality assessment (quadruplicate measurements) and average larval size determination (15 larvae measured). Larval counts and sizing were determined under an inverted microscope at ×40 magnification. The feeding trial was terminated on day 16 post-fertilization.

### Statistical analysis

Specific growth rate, *μ* (expressed in divisions per day, d^−1^) was calculated across the linear part of the ln-transformed growth curve according to Eq (1).

1 $$ \mu =\frac{3.322}{\left({t}_t-{t}_1\right)}\log \frac{N_t}{N_1} $$

With N_1_, the starting microalgal cell density (in cells mL^−1^) measured following inoculation at the start of the experiment (i.e., *t*_1_ in days); *N*_*t*_, the microalgal cell density at stationary phase, and *t*_*t*_, the time required to reach stationary phase cell density (Guillard 1973). Microalgal cell densities were quantified immediately following culture subsample extraction by flow-cytometry. Each culture medium treatment was performed in triplicate for bubbled experiments and in quadruplicate for static experiments. Specific growth rates are presented as means with associated 95% confidence interval.

Microalgal growth and larval performance data for the different treatments were compared by one-way analysis of variance (ANOVA; *p* < 0.05) using the *R* software. Results were reported at 95% confidence intervals.

## Results

### Treatment effects upon algal growth rate and stationary-phase cell density — Static cultures

The individual effects of NAC, F127, and F68 culture media enrichments upon the performance of *Chaetoceros calcitrans* and *Tisochrysis lutea* cultures grown in static cultivation flasks are reported in Table 1. Treatment effects upon algal culture performance are examined based upon changes in (i) specific growth rate in the exponential growth phase and (ii) stationary-phase cell density, relative to the control cultures. This initial screening revealed that supplementation of the culture medium with F127 increased specific growth rates of both *C. calcitrans* and *T. lutea*, with a more pronounced effect on the former (i.e., a doubling of specific growth rate for the diatom relative to a 16% increase in growth rate for the flagellate). Culture media enrichments with pluronic F68 had no statistically significant effect upon the specific growth rate of *C. calcitrans* and was, in fact, detrimental to the culture performance of *T. lutea*.

**Table 1:** Specific growth rate, stationary-phase cell density, and factorial increase relative to the *control* treatment (i.e., conventional F-medium) of *Chaetoceros calcitrans* and *Tisochrysis lutea* static cultures in response to supplementation of F127 (0.001% w/v), F68 (0.01% w/v), and NAC (1 mM) singly added to the culture medium. Specific growth rates are reported as mean division rates when averaged over the course of linear portion of the exponential growth phase of the cultures (i.e., 14 days and 12 days for *Chaetoceros calcitrans* and *Tisochrysis lutea*, respectively). Values are reported as means ± 95% confidence intervals, *n* = 4. ns: no significant difference (1-way ANOVA; *p* > 0.05). na: is not applicable

	*Chaetoceros Calcitrans*				*Tisochrysis lutea*			
	Specific growth rate (d^−1^)	Factorial increase relative to control	Stationary-phase cell density (cells mL^−1^)	Factorial increase relative to control	Specific growth rate (d^−1^)	Factorial increase relative to control	Stationary-phase cell density (cells mL^−1^)	Factorial increase relative to control
Control	0.17 ± 0.01	na	9.63E06 ± 3.49E05	na	0.27 ± 0.01	na	1.72E07 ± 2.27E06	na
NAC	0.21 ± 0.01	1.26	1.13E07 ± 2.99E05	1.17	0.26 ± 0.01	ns	1.31E07 ± 8.44E05	0.76
F127	0.37 ± 0.01	2.22	1.15E07 ± 1.18E05	1.19	0.31 ± 0.01	1.16	1.76E07 ± 2.37E6	1.02
F68	0.19 ± 0.03	ns	1.06E07 ± 9.96E05	ns	0.19 ± 0.01	0.73	1.57E07 ± 1.26E06	0.91

Addition of NAC to the culture media was beneficial for the performance of *C. calcitrans*, with a 26% increase in specific growth rate and 17% increase in stationary-phase cell density. No statistically significant effect in specific growth rate was observed for *T. lutea* cultures grown in NAC-enriched media.

### Treatment effects upon algal growth rate and stationary-phase cell density — Bubbled cultures

The effects of culture media enrichments with NAC and pluronic, singly or combined, on the culture development of the microalgal strains grown in laboratory-scale, bubbled photobioreactors were evaluated relative to the culture development of control cultures grown in F-medium. Screening of 25 treatments (electronic supplemental material-Table S1) revealed that algal culture performance response to supplementation with NAC and/or pluronic compounds was dependent upon (i) species, (ii) treatment composition, and (iii) treatment dosage. Final culture concentrations of NAC at or beyond 5 mM had detrimental effects upon the culture performance of all microalgal species investigated, often resulting in cell death following a 24-h exposure. A final culture concentration of 1 mM NAC was found to be optimal for maintaining or enhancing algal productivity, relative to the control, for all microalgal species examined in this study. For pluronic culture media additives, the optimal culture responses were found at 0.01% and 0.005% final concentration for pluronic F68 and F127, respectively, except for *P. costatum*, which responded negatively to pluronic medium addition independent of dosage. These pluronic dosages resulted in minimal foam formation at the culture surface. No algal cell clumping was observed resulting from pluronic-induced foaming throughout the course of the experiment. Addition of antifoam [10^−4^ % (w/v) final culture concentration] was found to neutralize culture foaming with no statistically significant effect upon culture performance.

**Fig. 1:**
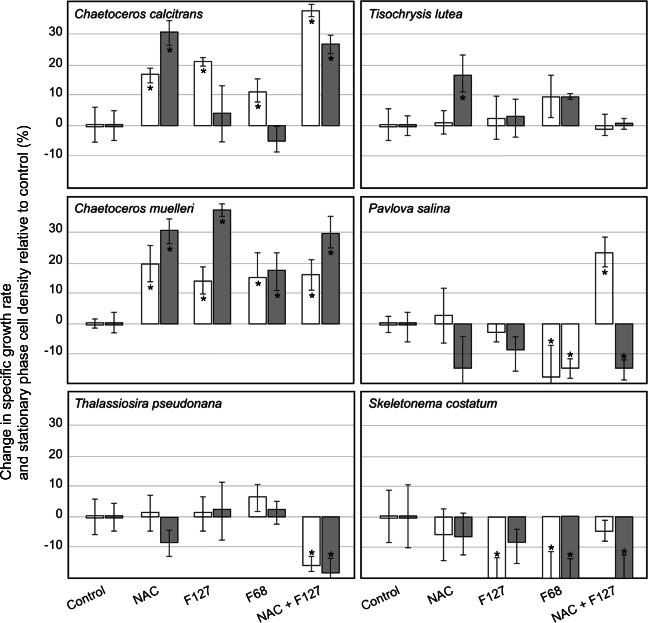
Percent change in specific growth rate (white bars) and stationary-phase density (grey bars) for six phytoplankton species exposed to single or combined NAC (1 mM) and pluronic [F127 (0.005% w/v) and F68 (0.01% w/v)] treatments in bubbled cultures. Error bars represent 95% confidence intervals (*n*=3). Significant differences relative to the control are notated with an asterisk (1-way ANOVA; *p* < 0.05)

Treatment effects upon algal culture performance at the optimal treatment dosage [1 mM for NAC, 0.01% and 0.005% (w/v) for F68 and F127, respectively, expressed as final culture concentration] across marine microalgae strains examined in this study are illustrated in Fig. 1. Treatment effects are reported as % changes in specific growth rate and final cell density relative to the control cultures. Single and combined NAC and pluronic treatments resulted in a statistically significant increase in the specific growth rate in both *Chaetoceros* species (Fig. 1). The stationary-phase cell density of *Chaetoceros* species increased following single and combined NAC and pluronic treatments, with the exception of single pluronic (F127 and F68) treatments in *C. calcitrans* cultures wherein no detectable change in stationary-phase cell density was observed. Pluronic medium additives had no significant effect upon the culture performance of *T. pseudonana* and *T. lutea* and were detrimental to *P. salina* and *S. costatum*. The NAC and combined NAC and F127 treatments were the most effective at amplifying the culture performance of *C. calcitrans*, with 16% and 38% increases in specific growth rate, and 30% and 26% increases in stationary-phase cell density, respectively. *C. muelleri* experienced an ~15% increase in specific growth rate with single and combined treatments including NAC, F127, and F68. The most significant increase in culture productivity was observed for *C. muelleri* cultures following F127 media supplementation (i.e., 14% and 36% increase in specific growth rate and stationary-phase density, respectively). Single NAC and pluronic culture medium additives had no significant effect upon the culture performance of *T. pseudonana*, and the combined NAC and F127 treatment resulted in ~20% decreases in specific growth rate and stationary-phase cell density. The culture performance of diatom *S. costatum* was degraded by NAC and pluronic medium additives (Fig. 1). Single pluronic and combined pluronic NAC media treatments had no significant effect upon the specific growth rate of *T. lutea*. A 16% increase in stationary-phase cell density was observed for the single NAC treatment in *T. lutea* cultures. Combined NAC and F127 media additions to *P. salina* cultures resulted in a 24% increase in specific growth rate but a 14% decrease in stationary-phase cell density (Fig. 1). Other investigated treatments had no significant effect upon the culture performance of *P. salina*.

### Effects of NAC and pluronic block polymer culture medium additives upon microalgal unsaturated fatty acid composition

**Fig. 2:**
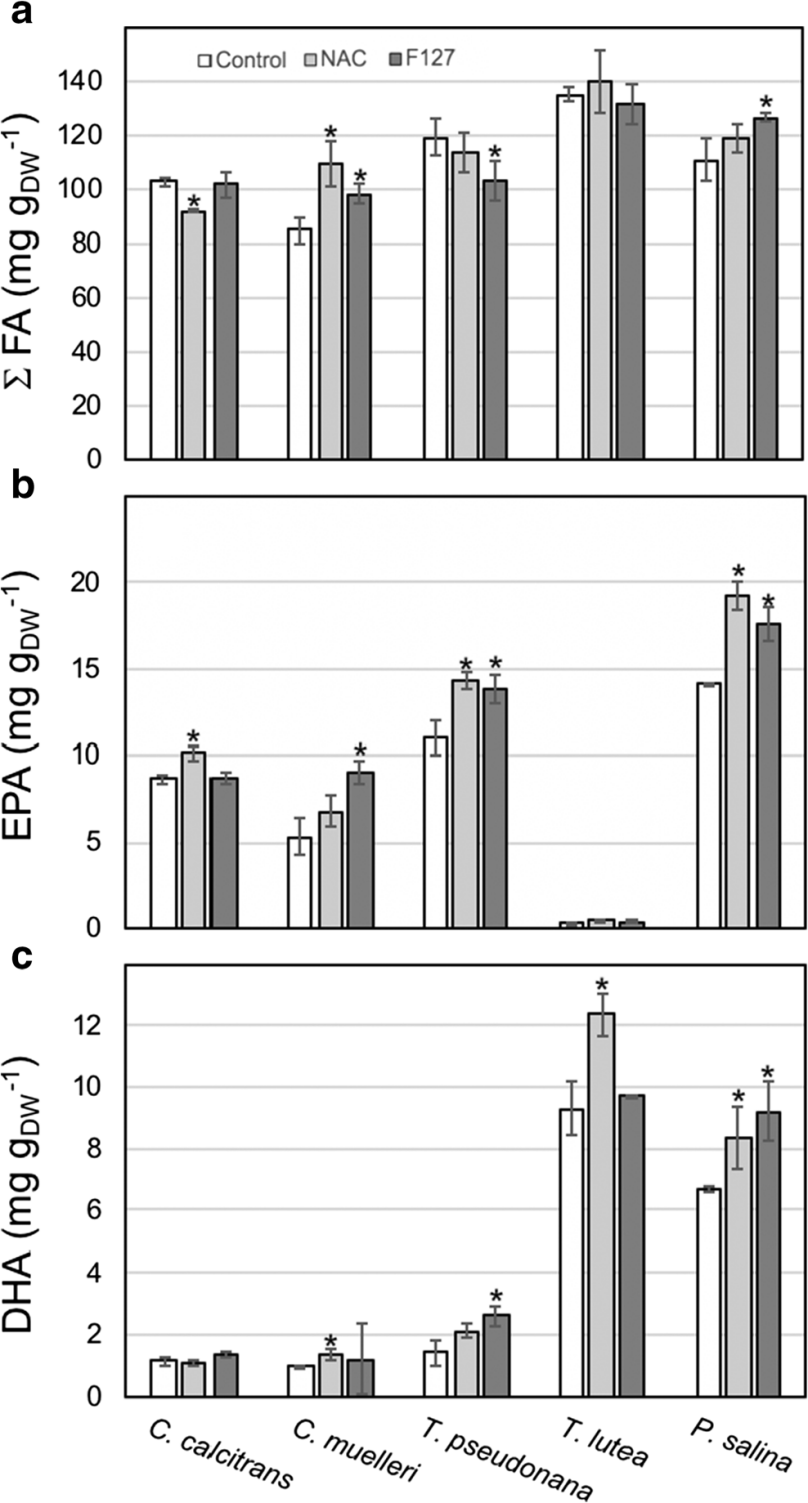
**a** Total fatty acids (FA) content, **b** DHA = docosahexaenoic acid, and **c** EPA = eicosapentaenoic acid for five phytoplankton species grown in NAC (1 mM) or F127 (0.005% w/v) supplemented F-enriched seawater in bubbled cultures. **a** through **c** expressed in mg fatty acid per gram dry weight (DW). Error bars represent 95% confidence intervals (*n*=3). Significant differences relative to the control are notated with an asterisk (1-way ANOVA; *p* < 0.05)

Total fatty acid content and essential omega-3 fatty acid docosahexaenoic acid (DHA) and eicosadienoic acid (EDA) fractions of algal biomass harvested at stationary-phase in bubbled culture flasks, expressed in mass fatty acid per mass dry weight, are illustrated on Fig. 2. Varied treatment responses were observed in the unsaturated fatty-acid profiles of algal biomass dependent on the microalgal species examined. Across diatom and flagellate species examined in this study, NAC and F127 culture medium additives had generally no significant effect upon algal biomass total fatty acid content. Statistically significant increases in total fatty acid content of 29% and 16% was observed for *C. muelleri*, when grown in a NAC- and F127-enriched media, respectively. Enrichment of the culture media with F127 resulted in a 22% increase in total fatty acid content of *P. salina* cultures. About 5 and 15% decreases in total fatty acid content were observed for *T. pseudonana* and *C. calcitrans* following NAC and F127 medium supplementation, respectively. Addition of NAC to the culture medium increased the DHA content of all species by 26–34%, except for *C. calcitrans* and *T. pseudonana* for which no statistically significant effect was observed. The EPA content of the microalgal species examined was not altered following NAC addition or showed a statistically significant increase of 16–36%, with the smallest increase observed in *C. calcitrans* and the largest in *P. salina*. The highest EPA and DHA content increase were observed in F127 culture medium supplementation in *C. muelleri* (70%) and *T. pseudonana* (85%). As with NAC addition, F127 supplementation had no effect upon the DHA content of *C. calcitrans*. No significant decrease in EPA or DHA was observed following single or combined NAC and pluronic treatments across marine microalgae species examined.

Given the beneficial treatment effects upon total fatty acid content, as well as DHA and EPA content in *C. muelleri*, we examined combined treatment effects upon the algal biomass fatty acid composition (Table 2). Combined NAC and F127 media supplementation in *C. muelleri* cultures resulted in a statistically significant increase in the total fatty acid content of 19% and 32% and 17% increases in DHA and EPA content, respectively.

**Table 2:** Total fatty acid and omega-3 fatty acid composition, expressed in mg fatty acid per gram dry weight, for *C. muelleri* following single and combined culture media addition of NAC (1 mM) and pluronic block polymers [F127 (0.005% w/v) and F68 (0.01% w/v)]. Values are reported as means ± 95% confidence intervals, *n* = 3. FA, total fatty acids content; DHA, docosahexaenoic acid; and EPA, eicosapentaenoic

	Control	NAC	F127	NAC + F127	NAC + P68
14:0	7.91 ± 0.72	10.00 ± 0.63	9.12 ± 0.42	10.22 ± 0.53	10.03 ± 0.34
14:1(*n*-5)	0.19 ± 0.02	0.21 ± 0.01	0.26 ± 0.03	0.38 ± 0.02	0.33 ± 0.02
15:0	0.96 ± 0.10	1.24 ± 0.07	0.88 ± 0.12	1.25 ± 0.09	1.19 ± 0.07
15:1(n-5)	0.18 ± 0.03	0.24 ± 0.02	0.18 ± 0.02	0.22 ± 0.01	0.22 ± 0.03
16:0	33.09 ± 1.12	32.60 ± 0.82	33.90 ± 0.82	33.73 ± 0.64	28.28 ± 0.50
16:1(n-7)	22.67 ± 1.13	33.62 ± 2.70	23.36 ± 0.72	26.91 ± 0.52	28.79 ± 0.60
17:0	0.11 ± 0.03	0.08 ± 0.02	0.07 ± 001	0.19 ± 0.02	0.08 ± 0.02
17:1(n-7)	0.05 ± 0.01	0.08 ± 0.02	0.13 ± 0.05	0.10 ± 0.02	0.09 ± 0.02
18:0	0.75 ± 0.04	0.58 ± 0.02	0.76 ± 0.08	0.69 ± 0.03	0.56 ± 0.06
18:1(n-9)	1.53 ± 0.13	1.04 ± 0.02	1.71 ± 0.01	1.37 ± 0.05	1.02 ± 0.05
18:1(n-7)	1.22 ± 0.14	2.35 ± 0.12	1.40 ± 0.08	2.22 ± 0.09	2.50 ± 0.11
18:2(n-6)-t	0.09 ± 0.01	0.22 ± 0.01	0.09 ± 0.03	0.19 ± 0.01	0.24 ± 0.02
18:2(n-6)-c	0.70 ± 0.04	0.51 ± 0.03	0.71 ± 0.01	0.63 ± 0.02	0.49 ± 0.03
19:0	0.08 ± 0.01	0.17 ± 0.03	0.15 ± 0.02	0.12 ± 0.01	0.14 ± 0.03
18:3(n-6)	0.04 ± 0.01	0.08 ± 0.02	0.06 ± 0.02	0.08 ± 0.02	0.08 ± 0.05
19:1(n-9)	1.81 ± 0.13	1.50 ± 0.72	1.73 ± 0.42	1.53 ± 0.32	1.22 ± 0.33
18:3(n-3)	0.10 ± 0.02	0.08 ± 0.01	0.05 ± 0.03	0.13 ± 0.01	0.07 ± 0.01
18:4(n-3)	1.32 ± 0.10	0.58 ± 0.07	1.23 ± 0.11	1.11 ± 0.05	1.12 ± 0.02
20:4(n-6)	1.38 ± 0.08	1.39 ± 0.02	1.35 ± 0.72	1.29 ± 0.03	1.09 ± 0.01
22:0	0.35 ± 0.01	0.24 ± 0.02	0.39 ± 0.02	0.43 ± 0.06	0.34 ± 0.02
20:5(n-3) [EPA]	5.38 ± 0.40	6.79 ± 0.10	9.07 ± 0.12	6.33 ± 0.23	7.25 ± 0.12
22:4(n-3)	0.46 ± 0.04	0.50 ± 0.10	0.49 ± 0.12	0.54 ± 0.07	0.44 ± 0.02
22:6(n-3) [DHA]	0.99 ± 0.01	1.35 ± 0.11	1.19 ± 0.07	1.31 ± 0.07	1.30 ± 0.06
sum(n-3)	7.58 ± 0.63	8.81 ± 0.72	8.16 ± 0.50	8.33 ± 0.83	9.22 ± 0.47
sum(n-6)	2.26 ± 0.30	2.28 ± 0.30	2.40 ± 0.42	2.27 ± 0.31	2.00 ± 0.12
total FA	86.93 ± 2.41	109.32 ± 3.42	98.15 ± 2.76	103.98 ± 2.32	100.32 ± 3.12

### Effects of NAC and pluronic block polymer culture medium additives upon algal TEP release

**Fig. 3:**
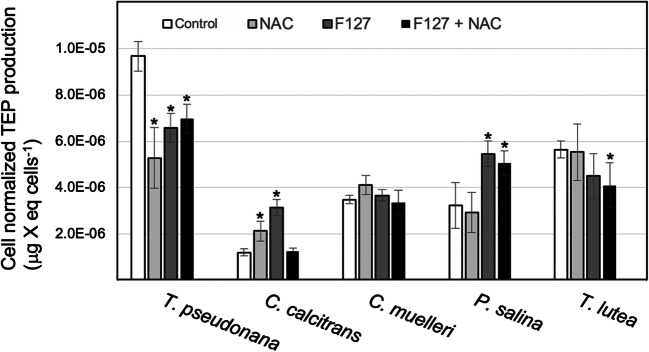
Cell-normalized average abundance of transparent exopolymer particles (TEP) in microalgae (bubbled) cultures following NAC (1 mM) and/or F127 (0.005% w/v) supplementation of the culture media. TEP abundances are expressed in mass xanthan (X) gum. Error bars represent 95% confidence intervals (*n*=3). Significant differences relative to the control are notated with an asterisk (1-way ANOVA; *p* < 0.05)

Cell-normalized TEP abundances, expressed in μg X eq cell^−1^, quantified at stationary-phase for marine microalgae species grown in NAC- and/or pluronic-enriched culture media are reported in Fig. 3. Of all marine microalgal species examined, cultures of *C. calcitrans* and *T. pseudonana*, respectively, exhibited the lowest (ranging between 1.22 and 3.15 μg X eq cells^−1^) and highest (ranging 5.28–9.67 μg X eq cells^−1^) cell-normalized extracellular TEP release, independent of treatment. No specific pattern in TEP production was observed for microalgae cultures grown in NAC- or F127-enriched media. For *T. pseudonana* cultures, single or combined NAC and F127 media supplementation resulted in 28–45% decreases in TEP production. No significant change in cell-normalized TEP abundance in *C. muelleri* cultures was observed following NAC and/or F127 media supplementation. A significant increase in TEP production per cell was observed for *C. calcitrans* (2.6 x) and *P. salina* (1.7 x) cultures grown in a F127-enriched media. Combined NAC and F127 media supplementation had no effect upon TEP production for *Chaetoceros* sp. and resulted in a significant decrease in TEP production in cultures of *T. pseudonana* and *T. lutea*, whereas the opposite effect was observed for *P. salina* cultures. Media supplementation with NAC had generally no effect upon cell-normalized TEP production, except for *T. pseudonana* and *C. calcitrans* cultures, which showed statistically significant decreases and increases, respectively.

### Oyster-larval performance

**Fig. 4:**
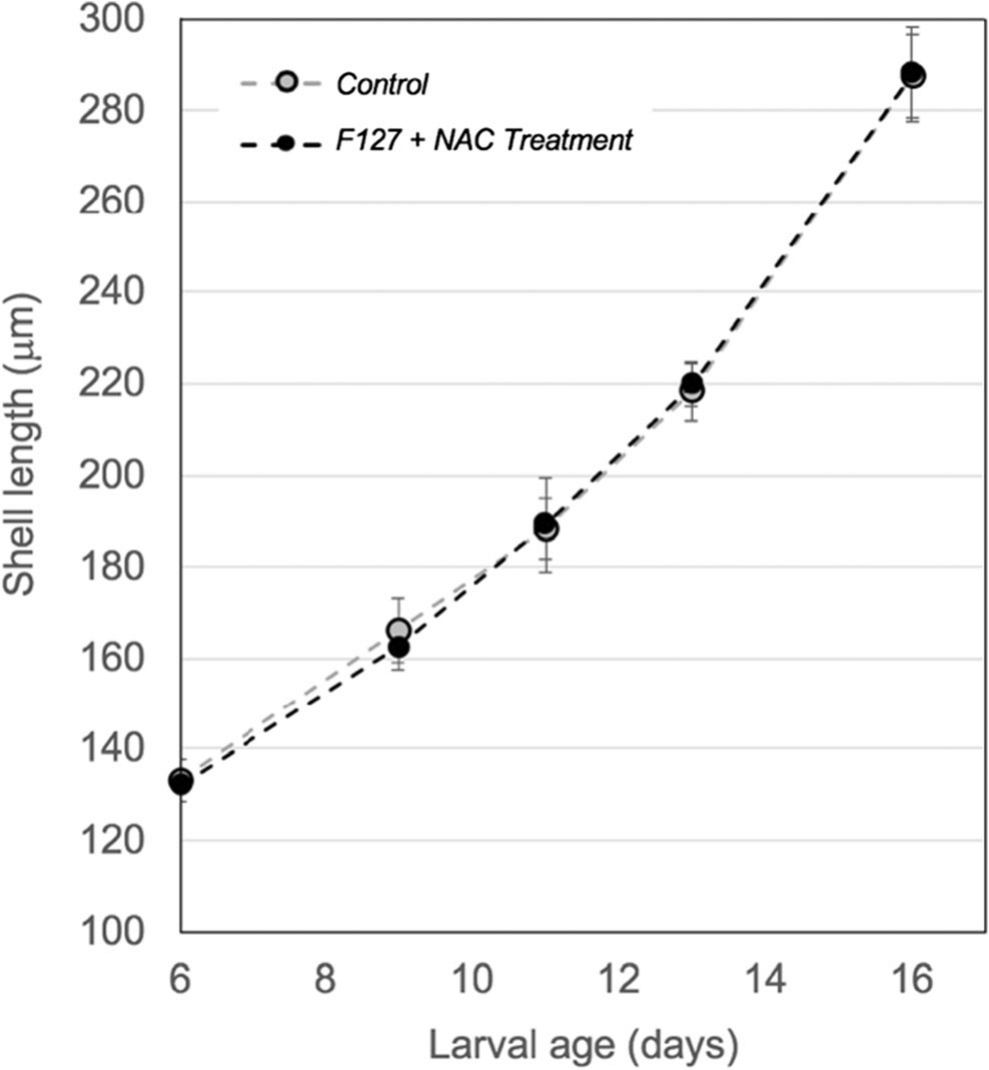
Shell growth of *Crassostrea gigas* larvae reared in 200-L static tanks on a diet consisting of equal proportions of *C. calcitrans*, *C. muelleri*, *P. salina*, and *T. lutea* grown in F-enriched seawater (black circles-*control*) and F-enriched seawater supplemented with Pluronic F127 [0.001% w/v] and NAC [1 mM] (*treatment*-grey circles). Error bars represent 95% confidence intervals based on 3 replicates

Larval development of Pacific oysters, *C. gigas*, fed a diet of microalgae grown in a NAC + F127-enriched F medium, compared with the development of larvae fed a conventional, F-medium-grown algal diet (*control*) is shown in Fig. 4. Following a 10-day feeding trial treatment and control diet fed larvae averaged 288 ± 7 μm and 284 ± 6 μm shell length, respectively, and a final 1 animal mL^−1^ stocking density. No statistically significant difference in larval growth, shell size, or mortality rate was observed between the treatment and control larval rearing tanks. Table 3 summarizes larval variables on the last day of the feeding trial (day 16 post fertilization and at day 10 of the feeding trial).

**Table 3:** *C. gigas* larvae at 16 days of larvae development (mean ± 95% confidence intervals, 3 replicates) after rearing on a 10-day F-enriched seawater grown algal diet (equal proportion of *C. calcitrans*, *C. muelleri*, *P. salina* and *T. lutea*) and F-medium supplemented with NAC (1 mM) and F127 (0.001% w/v).

Treatment	Shell length (μm)	Larvae concentration (larvae mL^−1^)	Proportion retained on 180-μm sieve (%)	% dead shells
F medium (*control*)	284 ± 6.3	1 ± 0.7	85 ± 5.0	4 ± 2.7
F medium + [NAC and F127)	288 ± 7.2	1 ± 0.5	87 ± 4.3	6 ± 1.5

## Discussion

### Overall findings

In a series of algal culture experiments in nutrient-replete (F media) and light-saturating conditions, we show culture medium supplementation with N-acetylcysteine and pluronic block polymers (F127 or F68) to be an effective strategy to enhance the algal yield of *C. calcitrans* and *C. muelleri* cultures. This supplementation, however, had generally no detectable effect upon the culture performance of haptophytes (i.e., *T. lutea* and *P. salina*) or *T. pseudonana*, and was detrimental to *S. costatum* culture development. Observed increases in specific growth rate were generally associated with increased in stationary-phase cell density. Depending up on the microalgal strain examined, culture media enrichments with NAC and/or pluronic resulted in increased algal biomass omega-3-fatty acid content.

We established a sensitivity range for pluronic and NAC culture medium additives and determined optimal treatment dosages resulting in increased culture performance of *Chaetoceros calcitrans*, *Chaetoceros muelleri*, *Pavlova salina*, and *Tisochrysis lutea* cultures-strains all used widely as live feeds in shellfish hatcheries.

Culture medium supplementation did not affect the quality of the algae for use as live feed in the culture of Pacific oyster larvae, thus demonstrating the applicability of the proposed approach in hatcheries. Below, several potential mechanisms underlying the enhanced culture performance with these medium additives are discussed.

### Role of N-acetylcysteine medium additives upon marine microalgal culture performance

Microalgae cultured phototrophically experience oxidative stress as a result of a wide variety of environmental factors [light, presence of pathogens, and nutrient deficiency] (Xie et al. 2019). Oxidative damage is associated with the generation of reactive oxygen species (ROS), including free radicals and peroxides (Janknegt et al. 2009). Cellular ROS build up is detrimental to growth, photosynthetic performance, and viability of the cultured cells (Milne et al. 2009; Rijstenbil 2003). Even under optimal culturing conditions, exposure to light and natural byproducts of photosynthesis inevitably contributes to elevated cellular ROS levels (Apel and Hirt 2004; Cirulis et al. 2013; Xie et al. 2019). Microalgae have developed a series of defense mechanisms to prevent ROS accumulation and to counteract oxidative damage, including antioxidant enzymes (e.g., glutathione reductase) and non-enzymatic antioxidant molecules [e.g., pigments] (Cirulis et al. 2013). Glutathione reductase and reduced glutathione (GSH) are two major components of the ascorbate-glutathione pathway, which play an essential role in protecting cells against oxidative damage and maintaining cellular redox balance (Gill et al. 2013). Despite a series of inherent mechanisms to scavenge and neutralize ROS, the microalgal natural antioxidant system is not sufficient to prevent all oxidative-stress-related damage (Cirulis et al. 2013). NAC has been suggested to offer direct protection against oxidative stress through the scavenging of oxidant species and hydroxyl radicals (Anbar and Neta 1967) and indirectly by raising intracellular concentrations of cysteine, which is metabolized to GSH. In our study, NAC addition to static and bubbled cultures resulted in increased specific growth rate (e.g., up to 20% in the case of *Chaetoceros muelleri*) for cultures belonging to the genus *Chaetoceros*, whereas no detectable change in culture productivity was observed for the other microalgae strains examined. Microalgal responses to excess irradiance are highly species-dependent and are a consequence of the synergy of several protection mechanisms and cell characteristics of each strain (Janknegt et al. 2009). Our experiments suggest culture media enrichments with NAC are effective to increase culture productivity of *Chaetoceros* spp., likely because of contributions of this compound to cellular homeostasis maintenance.

Increased stationary-phase cell density between 16 and 30% was observed for *Tisochrysis lutea*, *Chaetoceos muelleri*, and *Chaetoceros calcitrans* when grown in NAC-enriched culture media. Examination of the nitrogen mass balance within individual, experimental flasks shows that at stationary-phase, ~17% of dissolved nitrogen in the culture media remained unutilized by the microalgae, indicating light-limited conditions and, thus, relatively minimized light-induced effects of oxidative stressors upon the microalgae. In the culture of plants, NAC-induced rise in cellular GSH content results in not only enhanced stress tolerance and maintenance of cell redox homeostasis, but likely also enhanced metabolic function and cell-regulatory processes (Yan et al. 1995), expression of plant-defense-related genes (Ball et al. 2004), and plant growth and photochemical reactions (Jahan et al. 2014). Observed increase in stationary-phase cell density in the NAC-supplemented culture medium is, therefore, possibly attributable to NAC-induced changes in algal physiology combined with oxidative stress protection function. Comparison of nutrient-limited and light-limited cultures supplemented with NAC is needed to clarify the effects upon stationary-phase cell density.

Cellular ROS accumulation, when not effectively managed, results in lipid peroxidation, DNA strand breakage, and chlorophyll bleaching, collectively threatening cell viability (Farmer and Mueller 2013). Lipids (through peroxidation of unsaturated fatty acids in membranes), proteins (through denaturation), carbohydrates, and nucleic acids are the main cellular components most susceptible to damage by free radicals (Cirulis et al. 2013; Kochevar 1990). Across microalgal strains examined in this study, culture media enrichments with NAC had no significant effect upon algal total fatty acid content, except for *C. calcitrans* and *C. muelleri*, wherein statistically significant decrease and increase in the total fatty acid content of 15% and 29%, respectively, were found. Additionally, microalgae grown in NAC-enriched media had elevated DHA and EPA contents. These observations suggest NAC medium additives have a stimulating function for cellular DHA and EPA synthesis or act as DHA-and-EPA-specific protective agents against peroxidation. These observations have important implications for the production of microalgae for use as live feed in the aquaculture sector as these fatty acids are considered to be nutritionally essential for the growth and development of many invertebrates and marine larval fish (Langdon and Waldock 1981; Watanabe 1993)

NAC is used routinely in medical and agricultural fields for its antimicrobial properties against a variety of Gram-negative and Gram-positive bacteria (Muranaka et al. 2013; Zhao and Liu 2010). In hydroponic settings, NAC has been proposed as an effective additive for the control of plant pathogens (Muranaka et al. 2013). In conjunction with antibacterial properties, NAC is known to impede bacterial adhesion to glass surfaces, exhibit mucus-dissolving properties, and inhibit bacterial exopolysaccharide production (Muranaka et al. 2013; Olofsson et al. 2003; Perez-Giraldo et al. 1997). In the presented study, the bacteria-free nature of the microalgae cultures implies that measured TEP abundances are of algal origin. No significant decrease in algal TEP release was observed in NAC-enriched cultures, with the exception of *Thalassiosira pseudonana*, wherein NAC-treated cultures had about half the TEP abundance of the control. Our observations imply that NAC media treatments might not be as effective at limiting TEP production in microalgae cultures compared with bacteria-dominated systems. Algal TEP release often is linked to stress conditions and/or nutrient limitation (Corzo et al. 2000). In the case of *C. calcitrans*, although NAC supplementation resulted in enhanced culture productivity, elevated TEP production paired with a significant decrease in total fatty acid content, points to suboptimal NAC-induced protection mechanisms against biotic and abiotic environmental stressors and associated lipid peroxidation.

Our findings support the multifaceted chemical and biological properties of NAC and imply that NAC is a promising compound for enhancing the productivity of microalgal cultivation systems and improving the fatty acid profile of algal biomass, most likely through offering protection from oxidative stress and altering growth-promoting physiological functions of the cultured algae.

### Role of pluronic block polymer medium additives upon marine microalgal culture performance

Results presented in Fig. 2 and Table 1 show that culture media enrichments with pluronic F127 or F68 can be either beneficial or detrimental to the culture performance of marine microalgae species. Culture medium enriched in pluronic F127 (0.005% w/v) and F68 (0.01% w/v) enhanced specific growth rates and resulted in increased stationary-phase density of air-sparged cultures of *Chaetoceros muelleri* and *Chaetoceros calcitrans*. Pluronic medium additives had no detectable effect on the culture productivity of *T. pseudonana* or *T. lutea* and were detrimental to *Pavlova salina* and *Skeletonema costatum* in air-agitated culture conditions. Pluronic medium additives can inhibit cellular metabolism or reduce cell viability, as observed for the dinoflagellate *Protoceratium reticulatum* (Gallardo-Rodríguez et al. 2012; Rodríguez et al. 2009). Our experiments show that 0.01–0.001% (w/v) final pluronic concentrations, although 2–3 orders of magnitude lower relative to standard dosages used in the culture of plant and mammalian cells, is detrimental to *S. costatum* and *P. salina*. Further examination of culture medium enrichments with a lower pluronic dosage is required to determine if pluronic block polymers have toxic effects on these microalgae species at even lower dosage. In contrast, our experiments show that microalgae from the genus *Chaetoceros*, as well as *T. pseudonana* and *T. lutea*, responded positively to pluronic medium additives, with an optimal concentration range of 0.01–0.005% (w/v). The applied pluronic concentrations are approximately 1–3 orders of magnitude lower than what appears to be optimal for regulating growth of cultured plant, insect, and mammalian cells (Kumar et al. 1992; Tharmalingam et al. 2008; Wu et al. 1997). The shear-stress alleviating effects of pluronic medium additives on cultured human lymphocytes (Mizrahi 1975), animal (Bentley et al. 1989), insect (Wu et al. 1997), plant, and microbial cells (King et al. 1990; King et al. 1988; Zhao et al. 2016) involve manipulation of the cell membrane system through the formation of a surfactant-mediated protective layer (Handa-Corrigan et al. 1989; Lowe et al. 1993). Pluronic block polymers have inherent surface-active properties imparting a tendency to accumulate at interfaces (Wu et al. 1997). Hydrophobic interactions are likely the primary means by which pluronic additives adsorb to the cell membrane in aqueous cell suspensions. The surfactant hydrophobic PPO groups attach to hydrophobic sites on the cell surface, such as lipids and membrane proteins, while the hydrophilic portion interacts with water (Wu et al. 1997). As the hydrophobic sites of the cell membrane are covered by the surfactant, the cell surface hydrophobicity decreases or becomes hydrophilic, reducing cell adhesion to gas bubbles, which in turn confers increased cellular resistance to shear stress (Murhammer and Goochee 1990a). The protective capacity of pluronics for the cultivation of animal cells in sparged bioreactors appears to be dependent upon the hydrophilic-lipophilic balance (HLB) of the applied pluronic, with compounds exhibiting a higher hydrophobic character acting as more effective protective agent (Murhammer and Goochee 1990b; Wu et al. 1997). Enhanced microalgae growth in the presence of F127 relative to F68 medium additives in the present study is consistent with these observations. Pluronic F127 is a larger molecule (factor of 50 in size) with a larger hydrophobic block, relative to pluronic F68, and thus a more potent culture medium additive to protect cultured cells from shear-stress-induced damage (Banquy et al. 2016).

The surfactant properties of pluronic compounds have relevant applications in microalgal production, as hydrodynamic forces generated by air bubbles and turbulence in sparged bioreactors are key limiting factors for culture performance (Barbosa et al. 2003; Rodríguez et al. 2009; Wang and Lan 2018). Sources of shear stress differ depending upon the cultivation system (Wang and Lan 2018). In air-agitated cultures, shear stress is generated by fluid circulation, micro-eddies, and the rupturing of air bubbles at the culture surface (Leupold et al. 2013). The shear-sensitivity of microalgae is species-dependent and determined by cell wall physical properties, cell morphology, and the presence of flagella (Wang and Lan 2018). Green algae generally exhibit the highest tolerance to shear stress, followed by cyanobacteria, haptophytes, red algae, and diatoms (Michels et al. 2016). In the present study, beneficial pluronic-induced effects upon culture performance were recorded for the more shear-sensitive *Chaetoceros* species, while no detectable effect was recorded for *Tisochrysis lutea*, independent of the pluronic compound utilized. Diatoms are characterized by a rigid cell wall composed of amorphous silica (Parkinson and Gordon 1999). A degree of variation in the rigidity and flexibility of algal cells is observed across diatoms species depending upon mechanical properties and morphology of the cells. More-rigid cells that are characteristic of *Chaetoceros* spp. experience larger shear forces compared with more flexible cells such as *Thalassiosira.* spp. (Hønsvall et al. 2016; Karp-Boss and Jumars 1998). The inherent lower tolerance to shear stress of *Chaetoceros* spp. might explain the positive pluronic treatment responses observed for *C. calcitrans* and C. *muelleri* relative to other haptophytes and diatom species investigated in this study. Based upon the effectiveness of pluronic medium additives in shear-stress mitigation for a wide variety of shear-sensitive cells (Michaels et al. 1995; Wu et al. 1997), we extrapolate that our empirical observation of enhanced algal culture performance in the presence of pluronic medium additives is likely the result of enhanced resilience to shear stress. Our observations suggest that pluronic culture medium additives are most effective for the cultivation of shear-sensitive microalgae, such as diatoms, relative to less shear-stress-sensitive species.

In addition to inhibiting cell growth, hydrodynamic shear forces can result in cellular oxidative stress causing peroxidation of membrane lipids (Gallardo-Rodríguez et al. 2012). In the present study, the total fatty acid contents of microalgae examined were unaffected or showed a statistically significant increase, with the exception of *T. pseudonana*. In all microalgae examined, F127 media supplementation had no detectable to beneficial effect upon algal DHA and EPA contents. The observed alteration of the fatty acid composition of algal biomass in the presence of pluronic culture medium additives is likely a product of minimized lipid peroxidation as a result of alleviated shear stress.

For several of the successful treatments, addition of pluronic block polymers resulted in enhanced specific growth rate and stationary-phase density in both sparged and non-sparged cultures. Observation of enhanced culture productivity in non-sparged cultures implies a biostimulating effect of these media additives beyond the known shear protection function. Similar growth-stimulating effects have been observed for plant cells in suspension culture when non-ionic surfactants, such as F68 and F127, were present in the culture media. Supplementation of culture medium with 0.0001–0.5% w/v of pluronic F68 stimulated shoot production from the petioles of *Corchorus capsularis* (white jute shrub) cotyledons, stimulated roots and callus production of transformed roots *Solanum dulcamara* (weed), and prolonged the time required prior to subculturing to prevent plant necrosis (Khatun et al. 1993; Kumar et al. 1992). Further, pluronics are known to exhibit a broad spectrum of biological response-modifying functions widely used in the pharmaceutical and medical field to enhance drug transport across cellular barriers (such as polarized intestinal epithelial cells and brain cells), enhancing gene transfer and activating the transcription of genes (Batrakova and Kabanov 2002). These effects are most apparent at pluronic concentrations below the critical micellar concentration (CMC), suggesting that unimers (i.e., single block copolymer molecules) are the main agents responsible for biological modifying properties. It is implied that a crucial role of unimers lies in their ability to incorporate and translocate across cellular membranes (Batrakova et al. 2001; Batrakova and Kabanov 2002). Applied pluronic dosages in the reported culture experiments lie below the CMC, implying that a variety of biological-response-modifying mechanisms that modulate cell physiology are likely occurring. For instance, our observation of increased microalgal culture productivity and enhanced omega-3-fatty acid profile in pluronic F127-enriched culture medium enrichment in both bubbled and non-bubbled experiments implies that other growth-promoting processes and/or enhanced cell membrane integrity protection mechanisms than surfactant properties are at play.

### Synergy between pluronic block polymers and N-Acetylcysteine

Combined NAC and F127 culture medium treatments were effective at enhancing algal culture performance of *Chaetoceros* species, generally elevating the total fatty acid content and DHA and EDA contents of algal biomass without increasing TEP production. These observations point to possible synergistic interactions between both compounds or additive effects of these two culture-medium constituents upon culture productivity. Previous experimental work reported a synergistic effect of NAC in combination with other antimicrobial substances [e.g., Ciprofloxacin] (Goldstein 2008; Zhao et al. 2016).

An increase in the production of reactive oxygen species (ROS) within shear-sensitive microalgal cells has been reported in response to hydrodynamic forces, resulting in peroxidation of cellular lipids and ultimately cell damage (Gallardo-Rodríguez et al. 2012). Addition of pluronic F127 at a concentration below the CMC in the algal culture experiments implies that F127 unimers might act as membrane-permeable carriers of NAC molecules, facilitating the delivery of antioxidant defense potential within the cell while providing beneficial surfactant-induced membrane system alterations. The conjunction of biological functions provided by the simultaneous presence of pluronics and NAC, or other antibacterial or growth promoting compounds in the algae culture media, opens promising possibilities for future studies aimed at enhancing culture performance and stability of microalgal cultivation platforms.

### Applications of NAC and pluronic block polymers in the production of live algal feeds

A feeding trial with *Crasosstrea gigas* larvae confirmed that algae grown with F127 and NAC supplementation were ingested and metabolized to the same degree as the control (i.e., F-medium-grown algae). Microalgae grown in this experimental culture medium also exhibited a nutritional profile enriched in omega-3 fatty acids relative to the control. As live food, the nutritional composition of algae, including the lipid [particularly n-3 polyunsaturated fatty acids (DHA and EDA)) and protein contents, is essential to the growth and development of larval and juvenile fish, molluscs, and crustaceans (Jónasdóttir et al. 2009; Langdon and Waldock 1981; Persoone and Claus 1980; Watanabe 1993; Wikfors et al. 1992). Improved algal nutritional profile resulting from NAC and F127 culture medium enrichments may be beneficial for hatchery productivity, in terms of survival and growth in subsequent nursery and grow out phases. Although we did not test the modified algae in rotifer or copepod cultures, it is possible that such algae display beneficial properties for use in fish hatcheries as well.

Mass production of live algal food under outdoor or greenhouse conditions remains a major bottleneck in the current production of shellfish spat (Persoone and Claus 1980). Inclusion of NAC and pluronic medium additives represents an easily implementable method to enhance the production capacity of certain marine microalgae species of relevance to aquaculture hatcheries. NAC and pluronic compounds are both non-toxic and biodegradable, making them sustainable to include in current algal medium formulations.

This study demonstrates that addition of NAC and pluronic F127 to current culture media formulations is an operationally simplistic approach to increase algal growth rates and omega-3-fatty acid content of marine microalgal species widely cultivated as live feed (*Chaetoceros* spp., *Pavlova salina*, and *Tisochrysis lutea*) for the rearing of aquatic organisms, particularly in the production of bivalve spat. The optimal treatment dosage across marine microalgal species examined is 1 mM for NAC, 0.01% and 0.005% (w/v) for pluronic F68 and F127, respectively, expressed as final culture concentration. Addition of the relatively more hydrophobic F127 pluronic to the culture media was generally more effective at enhancing algal culture performance (specific growth rate and stationary-phase cell density) compared with pluronic F68. Enhanced protection against oxidative damage and increased resistance of the cell membrane to shear stress are proposed as the main mechanisms governing the observed increase in microalgae growth and final culture cell density for the cultivation of marine microalgal species in aerated photobioreactors. The proposed culture medium additives likely also result in a range of beneficial physiological function modifications that may provide some degree of protection against bacterial pathogens and/or limit algal extracellular polymeric substance production. Considering the present and future potential of microalgae-based industries, especially in a context of bio-economy and global sustainability, research and development efforts geared toward enhancing microalgal biomass productivity are fundamental (Wijffels et al. 2010, Khan et al. 2018). Further exploration of the biological and chemical effects of NAC and pluronic culture medium enrichments, as well as an examination of the scalability of our observations to commercial production, will be valuable to realize the full potential of these medium additives to enhance the production efficiency of microalgal cultivation systems.

## Supplementary Information

ESM 1 (PDF 179 kb)

## Data Availability

All data generated or analyzed during this study are included in this published article (and its supplementary information files).
