# Supplementary material for: Effect of pluronic block polymers and N-acetylcysteine culture media additives on growth rate and fatty acid composition of six marine microalgae species
Source: Appl Microbiol Biotechnol. 2021 Feb 12;105(5):2139–56. doi: 10.1007/s00253-021-11147-8 (PMC7907027; doi:10.1007/s00253-021-11147-8)
Supplement: Supplementary file 1 — (PDF 179 kb) [file 253_2021_11147_MOESM1_ESM.pdf]

Electronic Supplemental Material - Effect of pluronic block polymers and N-acetylcysteine culture media additives on growth rate and fatty acid composition of six marine microalgae species

Justine Sauvage[1], Gary H. Wikfors[2], Xiaoxu Li[3], Mark Gluis[3], Nancy Nevejan[4], Koen Sabbe[5], and Alyssa Joyce[1*]

[1] Department of Marine Science, University of Gothenburg, Gothenburg, Sweden

[2] NOAA Fisheries Service (NMFS) Northeast Fisheries Science Center, Milford, CT 06460, USA

[3] South Australian Research and Development Institute, Primary Industries and Regions (PIRSA) Aquatic Sciences Centre, West Beach, SA, Australia

[4] Laboratory of Aquaculture & Artemia Reference Center, Faculty of Bioscience Engineering, Ghent University, Ghent, Belgium.

[5] Laboratory of Protistology & Aquatic Ecology, Faculty of Sciences, Ghent University, Ghent, Belgium.

* Corresponding author: alyssa.joyce@gu.se; Carl Skottbergsgata 22 B, 413 19 Göteborg, Sweden.

Journal: Applied Microbiology and Biotechnology; Prof. Alexander Steinbüchel, editor-in-chief.

Manuscript submitted as an Original article (Methods and protocols)

Treatment number	culture environment	NAC dosage (mM)	Pluronic	Pluronic dosage (% w/v)	Antifoam (% w/v)	Laboratory
1	static flasks	*control*				NOAA Milford Lab.
2	static flasks	1				NOAA Milford Lab.
3	static flasks		F127	0.001		NOAA Milford Lab.
4	static flasks		F68	0.01		NOAA Milford Lab.

1	bubbled flasks	*control*				SARDI-PIRSA
2	bubbled flasks				0.01	SARDI-PIRSA
3	bubbled flasks				0.001	SARDI-PIRSA
4	bubbled flasks				0.0001	SARDI-PIRSA
5	bubbled flasks	0.1				SARDI-PIRSA
6	bubbled flasks	1				SARDI-PIRSA
7	bubbled flasks	5				SARDI-PIRSA
8	bubbled flasks		F127	0.01		SARDI-PIRSA
9	bubbled flasks		F127	0.001		SARDI-PIRSA
10	bubbled flasks		F127	0.005		SARDI-PIRSA
11	bubbled flasks		F68	0.1		SARDI-PIRSA
12	bubbled flasks		F68	0.01		SARDI-PIRSA
13	bubbled flasks		F68	0.001		SARDI-PIRSA
14	bubbled flasks	0.1	F127	0.01		SARDI-PIRSA
15	bubbled flasks	1	F127	0.001		SARDI-PIRSA
16	bubbled flasks	5	F127	0.005		SARDI-PIRSA
17	bubbled flasks	0.1	F68	0.1		SARDI-PIRSA
18	bubbled flasks	1	F68	0.01		SARDI-PIRSA
19	bubbled flasks	5	F68	0.001		SARDI-PIRSA
20	bubbled flasks	0.1	F127	0.01	0.0001	SARDI-PIRSA
21	bubbled flasks	1	F127	0.001	0.0001	SARDI-PIRSA
22	bubbled flasks	5	F127	0.005	0.0001	SARDI-PIRSA
23	bubbled flasks	0.1	F68	0.1	0.0001	SARDI-PIRSA
24	bubbled flasks	1	F68	0.01	0.0001	SARDI-PIRSA
25	bubbled flasks	5	F68	0.001	0.0001	SARDI-PIRSA

table S1. List and composition of culture media treatments examined in this study.
